# Wrist-Worn Wearables for Monitoring Heart Rate and Energy Expenditure While Sitting or Performing Light-to-Vigorous Physical Activity: Validation Study

**DOI:** 10.2196/16716

**Published:** 2020-05-06

**Authors:** Peter Düking, Laura Giessing, Marie Ottilie Frenkel, Karsten Koehler, Hans-Christer Holmberg, Billy Sperlich

**Affiliations:** 1 Integrative and Experimental Exercise Science Department of Sport Science University of Würzburg Würzburg Germany; 2 Department of Sport Psychology Institute for Sport and Sport Sciences Heidelberg University Heidelberg Germany; 3 Department of Sport and Health Science Technical University of Munich Munich Germany; 4 Department of Health Sciences Mid Sweden University Östersund Sweden; 5 Department of Physiology and Pharmacology Karolinska Institute Stockholm Sweden

**Keywords:** cardiorespiratory fitness, innovation, smartwatch, technology, wearable, digital health

## Abstract

**Background:**

Physical activity reduces the incidences of noncommunicable diseases, obesity, and mortality, but an inactive lifestyle is becoming increasingly common. Innovative approaches to monitor and promote physical activity are warranted. While individual monitoring of physical activity aids in the design of effective interventions to enhance physical activity, a basic prerequisite is that the monitoring devices exhibit high validity.

**Objective:**

Our goal was to assess the validity of monitoring heart rate (HR) and energy expenditure (EE) while sitting or performing light-to-vigorous physical activity with 4 popular wrist-worn wearables (Apple Watch Series 4, Polar Vantage V, Garmin Fenix 5, and Fitbit Versa).

**Methods:**

While wearing the 4 different wearables, 25 individuals performed 5 minutes each of sitting, walking, and running at different velocities (ie, 1.1 m/s, 1.9 m/s, 2.7 m/s, 3.6 m/s, and 4.1 m/s), as well as intermittent sprints. HR and EE were compared to common criterion measures: Polar-H7 chest belt for HR and indirect calorimetry for EE.

**Results:**

While monitoring HR at different exercise intensities, the standardized typical errors of the estimates were 0.09-0.62, 0.13-0.88, 0.62-1.24, and 0.47-1.94 for the Apple Watch Series 4, Polar Vantage V, Garmin Fenix 5, and Fitbit Versa, respectively. Depending on exercise intensity, the corresponding coefficients of variation were 0.9%-4.3%, 2.2%-6.7%, 2.9%-9.2%, and 4.1%-19.1%, respectively, for the 4 wearables. While monitoring EE at different exercise intensities, the standardized typical errors of the estimates were 0.34-1.84, 0.32-1.33, 0.46-4.86, and 0.41-1.65 for the Apple Watch Series 4, Polar Vantage V, Garmin Fenix 5, and Fitbit Versa, respectively. Depending on exercise intensity, the corresponding coefficients of variation were 13.5%-27.1%, 16.3%-28.0%, 15.9%-34.5%, and 8.0%-32.3%, respectively.

**Conclusions:**

The Apple Watch Series 4 provides the highest validity (ie, smallest error rates) when measuring HR while sitting or performing light-to-vigorous physical activity, followed by the Polar Vantage V, Garmin Fenix 5, and Fitbit Versa, in that order. The Apple Watch Series 4 and Polar Vantage V are suitable for valid HR measurements at the intensities tested, but HR data provided by the Garmin Fenix 5 and Fitbit Versa should be interpreted with caution due to higher error rates at certain intensities. None of the 4 wrist-worn wearables should be employed to monitor EE at the intensities and durations tested.

## Introduction

Physical activity reduces the incidences of noncommunicable diseases, obesity, and mortality, but, unfortunately, according to the World Health Organization (WHO), a sedentary lifestyle is becoming increasingly common, with approximately 23% of the adult population failing to meet physical activity guidelines [1-3]. Accordingly, innovative approaches to promote and monitor physical activity are urgently warranted, as indicated in the WHO’s global action plan [4]. While individual monitoring of physical activity aids in the design of effective interventions to enhance physical activity [5,6], a basic prerequisite is that the monitoring devices exhibit high validity.

Heart rate (HR) and energy expenditure (EE) are two key aspects of physical activity. HR reflects the intensity of physical activity [7,8], while monitoring EE is particularly helpful for individuals seeking to regulate their body mass or composition [9], since any imbalance between energy intake and EE may have negative consequences [10]. HR and EE vary widely between individuals, and careful monitoring is crucial to provide appropriate recommendations concerning physical activity and diet [10].

While several procedures for monitoring HR (eg, Holter monitors or chest belts) and EE (indirect calorimetry) are available, miniaturized sensors [11] potentially enable less restrictive monitoring. Utilization of data collected by miniaturized wearable sensors (wearables) to improve health and fitness is a current worldwide trend [12] that offers new opportunities for designing individualized interventions concerning physical activity [13]. Theoretically, wearables allow extensive monitoring of parameters related to physical activity over prolonged periods [14]. Rigorous validation of wearable sensors is paramount since insurance companies encourage and promote monitoring (with wearables representing a major component of this strategy) [15], the WHO aims to endorse digital health (including wearables) [16], and in Germany, state laws already permit physicians to prescribe digital health solutions [17].

Wearable manufacturers claim to enable noninvasive and accurate monitoring of HR and EE [18]. The market for wearables designed to improve health and fitness is growing rapidly, and companies release new versions of their technology at least once each year, with older versions disappearing from the market. Projections for wrist-worn wearables alone estimate that 152.7 million such devices will be shipped in 2019, with a compound annual growth rate of 6.2% until 2023 [19]. However, the validity of most commercially available wearables has not been assessed across a range of exercise intensities by independent research institutions [18,20,21]. Consequently, while the potential health benefits of wearables are considerable, their validity must first be assured.

Accordingly, the current investigation was designed to assess the validity of 4 commercially available**,** high-tech, and popular wearable models for monitoring HR and EE while sitting or performing light-to-vigorous physical exercise.

## Methods

Our study protocol and data analysis were based on previous recommendations concerning the validation of the reliability of wearables for assessing parameters during physical activity [22].

### Participants

After being informed about the experimental procedures, 25 healthy participants (11 men, 14 women; mean age 26 years, SD 7 years; mean body height 174 cm, SD 10 cm; mean body mass 70.1 kg, SD 12.0 kg) of Caucasian origin gave their written consent to participate. This study was performed in accordance with the Declaration of Helsinki and approved by our institute’s ethical committee (Ethical approval number: EthikKomm-05/2019).

### Experimental Procedures

All participants visited the laboratory twice, with 3 days between visits, and tested 2 different wearables on each occasion. Environmental conditions were constant, with a temperature of 19.5 °C (SD 0.8 °C). Anthropometric data were collected during the first visit. Each wearable was attached to the wrist in the manner indicated by the manufacturer, and age, sex, height, and body mass were entered into the wearable’s software, along with information about whether the wearable was on the left or right wrist.

The wearables and the order in which they were worn during the first and second visits were chosen in a random fashion, resulting in 25 measurements with each wearable.

Each participant was monitored while sitting as well as during walking and running at different speeds (1.1 m/s, 1.9 m/s, 2.7 m/s, 3.6 m/s, and 4.1 m/s) for 5 minutes, interspersed with 5 minutes of standing still. All participants also performed 6 ~30-m sprints involving multiple changes in direction (ranging from 10° to 180°) on the SpeedCourt (GlobalSpeed GmbH, Hemsbach, Germany) [23]. This involved sprinting between 12 contact plates installed symmetrically in a 5.25 m by 5.25 m square on the floor. A software program designed a path consisting of the 6 30-m sprints (approximately 15 seconds per 30-m sprint), with a display indicating the contact plates that had to be touched [23].

**Figure 1:**
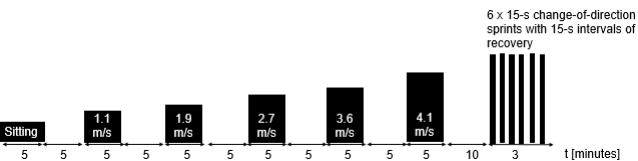
Schematic illustration of the periods during which each participant was monitored (black bars).

Figure 1 summarizes the sitting, walking, and running procedures.

#### Criterion Measures

A portable breath-by-breath gas analyzer (Metamax 3B, CORTEX Biophysik GmbH, Leipzig, Germany) employing standard algorithms for indirect calorimetry served as the criterion measure for EE. This system measures metabolic demands reliably [24] and has been used previously to assess the validity of wearables designed to monitor EE [25].

A Polar H7 chest belt, commonly employed for similar evaluations [26,27], was synchronized with the gas analyzer and served as the criterion measure for HR.

#### Wearables

The 4 tested wrist-worn wearables were Apple Watch Series 4, Version 5.1 (Apple Inc, Cupertino, CA); Polar Vantage V, Firmware 3.1.7 (Polar Electro Oy, Kempele, Finland); Garmin Fenix 5, Software 7.6 (Garmin, Olathe, KS); and Fitbit Versa, Version 32.33.1.30 (Fitbit Inc, San Francisco, CA).

All utilize photoplethysmography to monitor HR, but, to the best of our knowledge, information concerning the data used to calculate EE is not publicly available. Each wearable was positioned firmly, yet comfortably, on the wrist as in real life and as recommended by the manufacturers.

In the case of the Apple Watch Series 4, the “indoor walking” mode was selected for measurements while sitting or walking at 1.1 m/s; “running indoor” for speeds from 1.9 m/s to 4.1 m/s; and “HIIT” for the intermittent sprints. For the Polar Vantage V, the “Running (Treadmill)” mode was selected for all the monitoring periods, except for the intermittent sprints involving many and frequent changes in direction, for which “Soccer” was chosen. With the Garmin Fenix 5 and Fitbit Versa, the “Treadmill” mode was chosen for all monitoring periods.

All data were transmitted via Bluetooth and synchronized with the accompanying smartphone applications, in accordance with the manufacturers’ recommendations. For the Apple Watch Series 4, the raw data were exported to Microsoft Excel (Microsoft Corp, Redmond, WA) via the Apple Health App (Apple Inc, Cupertino, CA). In the cases of Polar, Garmin, and Fitbit, data were exported via specific buttons in the accompanying online software or collected directly from the software.

### Statistical Analysis

Statistical analysis was performed in accordance with previous recommendations, whenever applicable [22]. Prior to analysis, the data were log-transformed to avoid bias resulting from nonuniformity of error. All data were analyzed in custom-designed Microsoft Excel spreadsheets [28]. For each exercise, the standardized mean bias was calculated. As recommended and carried out previously, linear regression was employed to analyze validity [22,29]. The standardized mean bias, standardized typical error of the estimate (sTEE), coefficient of variation (CV), and Pearson’s product-moment correlation coefficient are all reported.

The sTEE, based on half the thresholds of the modified Cohen’s scale, was employed to assess validity: <0.1, trivial; 0.1-0.29, small; 0.3-0.59, moderate; 0.6-1.0, large; 1.0-2.0, very large; >2.0, extremely large [28]. Pearson’s *r* was utilized to evaluate the correlation between the criterion measure and wearable as follows: 0.45-0.69, very poor; 0.70-0.84, poor; 0.85-0.94, good; 0.95-0.994, very good; ≥0.995, excellent [30]. The 90% confidence limits (coefficient of variation [CV]) for the statistical parameters are also reported. Absolute errors were calculated based on these CVs and the mean value obtained by the criterion measure.

The level of physical activity was defined in terms of the metabolic equivalent (MET), with <3 MET indicating light, <6 MET medium, and >6 MET vigorous physical activity [31]. To define physical activity levels, the EE provided by the criterion measure was extrapolated to 1 hour and divided by the mean body weight of the participant.

## Results

### Heart rate

The mean HR, CV, Pearson’s *r*, and sTEE with 90% confidence limits and interpretations are summarized in Table 1.

**Table 1:** Analysis of the validity of heart rate measurements by wrist-worn wearables while sitting or walking/running at different intensities.

Level of activity (METs^a^), intensity		Apple Watch Series 4	Polar Vantage V	Garmin Fenix 5	Fitbit Versa
**Inactive (1.3), sitting**					
	Heart rate (bpm)^b^, mean (SD)	68.8 (11.7)			
	Standardized mean bias	0.03 (–0.02 to 0.07)	–0.06 (–0.11 to –0.02)	0.12 (–0.07 to 0.31)	–0.06 (–0.27 to 0.15)
	Pearson’s *r*	0.99 (0.99-1)	0.99 (0.98-1)	0.89 (0.77-0.95)	0.91 (0.77-0.96)
	Interpretation of Pearson’s *r*	Excellent	Excellent	Good	Good
	CV^c^ (%)	2 (1.6-2.6)	2.2 (1.8-2.9)	7.7 (6.1-10.7)	8 (6.1-12.1)
	sTEE^d^	0.12 (0.09-0.17)	0.13 (0.10-0.19)	0.63 (0.41-1.03)	0.47 (0.28-0.82)
	Interpretation of sTEE	Small	Small	Large	Moderate
**Light (3.5), 1.1 m/s**					
	Heart rate (bpm)^b^, mean (SD)	95.8 (25.0)			
	Standardized mean bias	0.01 (–0.07 to 0.09)	–0.07 (–0.32 to 0.17)	0.12 (–0.10 to 0.34)	–0.28 (–7.00 to 0.13)
	Pearson’s *r*	0.97 (0.95-0.99)	0.89 (0.79-0.94)	0.85 (0.70-0.93)	0.57 (0.31-0.70)
	Interpretation of Pearson’s *r*	Very good	Good	Good	Very poor
	CV (%)	2.9 (2.3-3.8)	5.5 (4.4-7.3)	5.8 (4.5-8.0)	9.6 (7.8-12.6)
	sTEE	0.23 (0.16-0.34)	0.54 (0.37-0.82)	0.62 (0.40-1.03)	1.43 (0.87-3.03)
	Interpretation of sTEE	Small	Moderate	Large	Very large
**Vigorous (6.6), 1.9 m/s**					
	Heart rate (bpm)^b^, mean (SD)	127 (19.4)			
	Standardized mean bias	–0.02 (–0.10 to 0.06)	–0.34 (–0.53 to –0.16)	0.06 (–0.17 to 0.29)	–0.05 (–0.34 to 0.24)
	Pearson’s *r*	0.97 (0.95-0.99)	0.91 (0.82-0.95)	0.83 (0.65-0.92)	0.54 (0.29-0.71)
	Interpretation of Pearson’s *r*	Very good	Good	Poor	Very poor
	CV (%)	2.9 (2.3-3.8)	5.4 (4.3-7.2)	9.2 (7.2-12.9)	19.1 (15.7-24.7)
	sTEE	0.23 (0.16-0.34)	0.46 (0.32-0.69)	0.68 (0.43-1.16)	1.58 (0.98-3.25)
	Interpretation of sTEE	Small	Moderate	Large	Very large
**Vigorous (9.9), 2.7 m/s**					
	Heart rate (bpm)^b^, mean (SD)	167 (16.5)			
	Standardized mean bias	–0.13 (–0.49 to 0.24)	–0.37 (–0.57 to –0.16)	–0.56 (–0.87 to –0.24)	–0.82 (–1.18 to –0.47)
	Pearson’s *r*	1 (0.99-1)	0.88 (0.78-0.94)	0.63 (0.34-0.81)	0.52 (0.27-0.70)
	Interpretation of Pearson’s *r*	Excellent	Good	Very poor	Very poor
	CV (%)	0.9 (0.7-1.2)	5.9 (4.8-7.9)	8.3 (6.6-11.4)	8.5 (7.0-11.0)
	sTEE	0.09 (0.06-0.12)	0.53 (0.36-0.81)	1.24 (0.74-2.73)	1.64 (1.01-3.59)
	Interpretation of sTEE	Trivial	Moderate	Very large	Very large
**Vigorous (10.4), 3.6 m/s**					
	Heart rate (bpm)^b^, mean (SD)	170 (15.3)			
	Standardized mean bias	0.02 (–0.09 to 0.14)	–0.75 (–1.05 to –0.46)	–0.40 (–0.60 to –0.19)	–1.17 (–1.47 to –0.87)
	Pearson’s *r*	0.94 (0.89-0.97)	0.86 (0.74-0.93)	0.82 (0.67-0.91)	0.82 (0.67-0.91)
	Interpretation of Pearson’s *r*	Good	Good	Poor	Poor
	CV (%)	3.0 (2.4-4.0)	4.9 (3.9-6.5)	8.9 (7.19-12.1)	4.1 (3.3-5.5)
	sTEE	0.35 (0.24-0.51)	0.59 (0.40-0.91)	0.69 (0.46-1.11)	0.70 (0.47-1.11)
	Interpretation of sTEE	Moderate	Moderate	Large	Large
**Vigorous (13.3), 4.1 m/s**					
	Heart rate (bpm)^b^, mean (SD)	177 (8.5)			
	Standardized mean bias	–0.27 (–0.51 to –0.03)	–0.72 (–0.95 to –0.49)	–1.47 (–1.88 to –1.06)	–2.06 (–3.17 to –0.95)
	Pearson’s *r*	0.85 (0.71-0.93)	0.89 (0.76-0.95)	0.82 (0.65-0.91)	0.68 (0.24-0.89)
	Interpretation of Pearson’s *r*	Good	Good	Poor	Very poor
	CV (%)	4.3 (3.4-5.8)	3.9 (3.0-5.6)	2.88 (2.28-3.96)	3.22 (2.34-5.35)
	sTEE	0.62 (0.41-1.00)	0.50 (0.31-0.84)	0.69 (0.44-1.17)	1.09 (0.52-4.13)
	Interpretation of sTEE	Large	Moderate	Large	Very large
**Vigorous (13.8), intermittent sprints**					
	Heart rate (bpm)^b^, mean (SD)	153 (14.7)			
	Standardized mean bias	0.12 (0.03 to 0.21)	–0.99 (–1.54 to –0.44)	–1.75 (–2.28 to –1.21)	–2.01 (–2.58 to –1.43)
	Pearson’s *r*	0.92 (0.85-0.96)	0.75 (0.53-0.88)	0.58 (0.28-0.78)	0.53 (0.15-0.77)
	Interpretation of Pearson’s *r*	Good	Poor	Very poor	Very poor
	CV (%)	3.5 (2.8-4.7)	6.7 (5.3-9.3)	8.4 (6.6-11.6)	9.0 (6.9-13.4)
	sTEE	0.38 (0.25-0.64)	0.88 (0.54-1.73)	1.44 (0.80-5.40)	1.94 (0.84-5.25)
	Interpretation of sTEE	Moderate	Large	Very large	Very large
**Vigorous (8.8), average of the values at all different intensities**					
	Heart rate (bpm)^b^, mean	137			
	Standardized mean bias	0.03	–0.47	–0.55	–0.92
	Pearson’s *r*	0.95	0.88	0.77	0.65
	Interpretation of Pearson’s *r*	Very good	Good	Poor	Very poor
	CV (%)	2.79	4.93	7.30	8.79
	sTEE	0.29	0.52	0.86	1.26
	Interpretation of sTEE	Moderate	Moderate	Large	Very large

^a^METs: metabolic equivalents.

^b^Measured according to the criterion measure.

^c^CV: coefficient of variation.

^d^sTEE: standardized typical error of the estimate.

**Figure 2:**
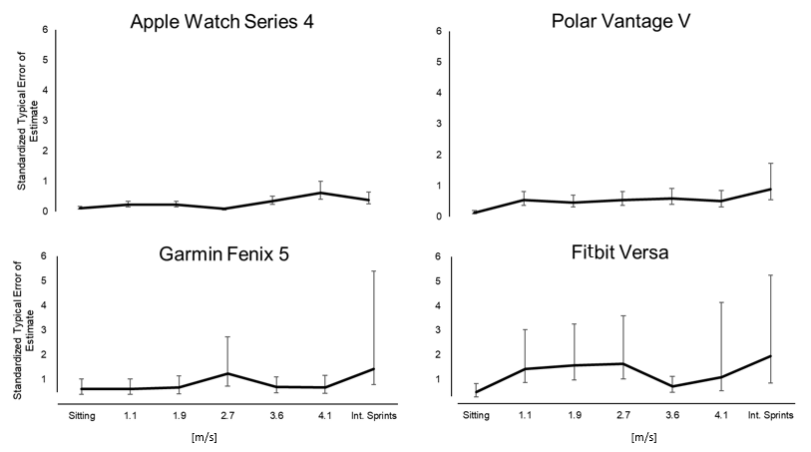
Standardized typical errors of the estimate (90% CI) for heart rate monitoring by the wearables while sitting or performing light-to-vigorous physical activity.

Figure 2 documents the sTEE for the HR values provided by the wearables at all exercise intensities.

For HR monitoring at the different intensities, the sTEE was 0.09-0.62, 0.13-0.88, 0.62-1.24, and 0.47-1.94 for the Apple Watch Series 4, Polar Vantage V, Garmin Fenix 5, and Fitbit Versa, respectively, with corresponding CVs of 0.9%-4.3%, 2.2%-6.7%, 2.88%-9.2%, and 4.1%-19.1%, respectively. The sTEE was less affected by intensity in the case of the Apple Watch Series 4 and Polar Vantage V devices than with the Garmin Fenix 5 and Fitbit Versa devices.

sTEE and CV peaked during the intermittent sprints for all the wearables except the Apple Watch Series 4.

### Energy Expenditure

The mean EE, CV, Pearson’s correlation coefficient, and sTEE with 90% confidence limits and interpretations are shown in Table 2.

**Figure 3:**
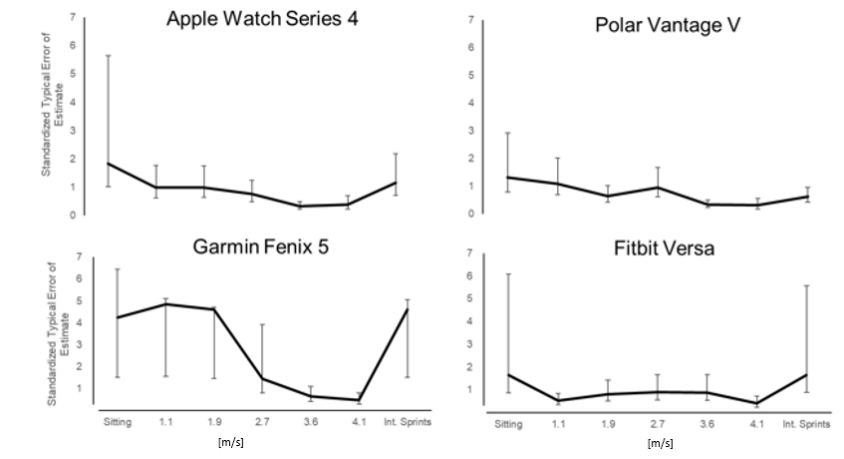
Standardized typical errors of the estimate (90% CI) for energy expenditure monitoring by the wearables while sitting or performing light-to-vigorous physical activity.

Figure 3 depicts the sTEE for the EE values provided by all 4 wearables during exercise at different intensities.

These sTEE values were 0.34-1.84, 0.32-1.33, 0.46-4.86, and 0.41-1.65 for the Apple Watch Series 4, Polar Vantage V, Garmin Fenix 5, and Fitbit Versa, respectively, with corresponding CVs of 13.5%-27.1%, 16.3%-28.0%, 15.9%-34.5%, and 8.0%-32.3%, respectively.

**Table 2:** Analysis of the validity of energy expenditure measurements by wrist-worn wearables while sitting and walking/running at different intensities.

Level of activity (METs^a^), intensity		Apple Watch Series 4	Polar Vantage V	Garmin Fenix 5	Fitbit Versa
**Inactive (1.3), sitting**					
	Energy expenditure (kcal/5 min)^b^, mean (SD)	7.6 (1.6)			
	Standardized mean bias	2.59 (2.25 to 2.94)	0.25 (–0.40 to 0.90)	1.74 (0.77 to 2.71)	–0.72 (–1.46 to 0.02)
	Pearson’s *r*	0.46 (0.16 to 0.68)	0.41 (0.10 to 0.65)	0.23 (–0.15 to 0.55)	0.52 (0.16 to 0.76)
	Interpretation of Pearson’s *r*	Very poor	**-**	**-**	Very poor
	CV^c^ (%)	26.6 (21.2-36.2)	28.0 (22.2-38.4)	20.9 (16.3-29.7)	17.1 (13.2-24.7)
	sTEE^d^	1.84 (1.02-5.64)	1.33 (0.79-2.94)	4.24 (1.51-6.46)	1.65 (0.87-6.09)
	Interpretation of sTEE	Very large	Very large	Extremely large	Very large
**Light (3.5), 1.1 m/s**					
	Energy expenditure (kcal/5 min)^b^, mean (SD)	20.6 (4.1)			
	Standardized mean bias	2.63 (2.23 to 2.03	1.29 (0.87 to 1.72)	–0.05 (–0.84 to 0.74)	4.16 (3.97 to 4.36)
	Pearson’s *r*	0.71 (0.49 to 0.85)	0.67 (0.44 to 0.82)	0.20 (–0.19 to 0.54)	0.88 (0.76 to 0.94)
	Interpretation of Pearson’s *r*	Poor	Very poor	-	Good
	CV (%)	15.1 (12.0-20.5)	16.3 (13.1-22.1)	16.8 (13.1-24.0)	8.0 (6.3-11.2)
	sTEE	0.99 (0.63-1.77)	1.10 (0.70-2.03)	4.86 (1.56-5.11)	0.53 (0.35-0.85)
	Interpretation of sTEE	Large	Very large	Extremely large	Moderate
**Vigorous (6.6), 1.9 m/s**					
	Energy expenditure (kcal/5 min)^b^, mean (SD)	38.3 (6.5)			
	Standardized mean bias	1.58 (1.27 to 1.90)	0.27 (–0.18 to 0.71)	–1.15 (–2.01 to –0.29)	0.88 (0.56 to 1.20)
	Pearson’s *r*	0.71 (0.49 to 0.84)	0.49 (0.18 to 0.7)	0.21 (–0.21 to 0.56)	0.78 (0.57 to 0.89)
	Interpretation of Pearson’s *r*	Poor	Very poor	-	Poor
	CV (%)	13.5 (10.8-18.1)	17.1 (13.7-23.1)	15.9 (12.2-23.3)	11.2 (8.8-15.7)
	sTEE	0.99 (0.64-1.76)	0.65 (0.43-1.02)	4.62 (1.46-4.73)	0.81 (0.51-1.44)
	Interpretation of sTEE	Large	Large	Extremely large	Large
**Vigorous (9.9), 2.7 m/s**					
	Energy expenditure (kcal/5 min)^b^, mean (SD)	57.8 (11.0)			
	Standardized mean bias	0.79 (0.56 to 1.02)	–0.09 (–0.39 to 0.2)	–0.04 (–0.45 to 0.37)	–0.06 (–0.44 to 0.32)
	Pearson’s *r*	0.80 (0.62 to 0.90)	0.72 (0.51 to 0.85)	0.57 (0.25 to 0.78)	0.74 (0.51 to 0.87)
	Interpretation of Pearson’s *r*	Poor	Poor	Very poor	Poor
	CV (%)	19.0 (15.1-26.2)	21.9 (17.5-29.8)	17.1 (13.3-24.4)	14.1 (11-19.8)
	sTEE	0.76 (0.50-1.25)	0.97 (0.62-1.68)	1.43 (0.80-3.91)	0.90 (0.50-1.67)
	Interpretation of sTEE	Large	Large	Very large	Large
**Vigorous (10.4), 3.6 m/s**					
	Energy expenditure (kcal/5 min)^b^, mean (SD)	60.5 (26.7)			
	Standardized mean bias	0.32 (0.19 to 0.45)	–0.05 (–0.18 to 0.08)	0.19 (–0.10 to 0.48)	–0.06 (–0.37 to 0.24)
	Pearson’s *r*	0.95 (0.89 to 0.97)	0.95 (0.89 to 0.97)	0.84 (0.68 to 0.92)	0.76 (0.52 to 0.88)
	Interpretation of Pearson’s *r*	Very good	Very good	Poor	Poor
	CV (%)	20.3 (16.0-28.3)	20.7 (16.4-28.6)	34.5 (26.4-50.8)	32.3 (24.6-48)
	sTEE	0.34 (0.23-0.50)	0.34 (0.24-0.51)	0.64 (0.41-1.09)	0.87 (0.53-1.65)
	Interpretation of sTEE	Moderate	Moderate	Large	Large
**Vigorous (13.3), 4.1 m/s**					
	Energy expenditure (kcal/5 min)^b^, mean (SD)	77.8 (46.6)			
	Standardized mean bias	0.34 (0.13 to 0.54)	–0.11 (–0.28 to 0.05)	0.25 (–0.06 to 0.55)	0.13 (–0.09 to 0.34)
	Pearson’s *r*	0.93 (0.82 to 0.98)	0.95 (0.87 to 0.98)	0.91 (0.78 to 0.96)	0.92 (0.81 to 0.97)
	Interpretation of Pearson’s *r*	Good	Very good	Good	Good
	CV (%)	27.1 (19.6-45.1)	22.7 (16.5-37.3)	33.1 (24.3-52.9)	29.9 (21.8-48.6)
	sTEE	0.39 (0.23-0.71)	0.32 (0.19-0.57)	0.46 (0.28-0.80)	0.41 (0.24-0.72)
	Interpretation of sTEE	Moderate	Moderate	Moderate	Moderate
**Vigorous (13.8), intermittent sprints**					
	Energy expenditure (kcal/5 min)^b^, mean (SD)	80.4 (15.6)			
	Standardized mean bias	1.83 (1.52 to 2.13)	0.23 (0.04 to 0.42)	–0.82 (–1.78 to 0.14)	–1.25 (–1.83 to –0.67)
	Pearson’s *r*	0.66 (0.41 to 0.81)	0.85 (0.72 to 0.92)	0.21 (–0.19 to 0.56)	0.42 (0.06 to 0.68)
	Interpretation of Pearson’s *r*	Very poor	Good	-	-
	CV (%)	25.4 (20.2-34.7)	17.5 (14.0-23.6)	17.9 (13.8-25.9)	20.8 (16.2-29.6)
	sTEE	1.15 (0.72-2.19)	0.63 (0.43-0.97)	4.62 (1.50-5.05)	1.64 (0.88-5.57)
	Interpretation of sTEE	Very large	Large	Extremely large	Very large
**Vigorous (8.8), average of the values at all different intensities**					
	Energy expenditure (kcal/5 min)^b^, mean	49.0			
	Standardized mean bias	1.44	0.26	0.02	0.44
	Pearson’s *r*	0.75	0.72	0.45	0.72
	Interpretation of Pearson’s *r*	Poor	Poor	Very poor	Poor
	CV (%)	21.0	20.6	22.3	19.1
	sTEE	0.92	0.76	2.98	0.97
	Interpretation of sTEE	Large	Large	Extremely large	Large

^a^METs: metabolic equivalents.

^b^Measured according to the criterion measure.

^c^CV: coefficient of variation.

^d^sTEE: standardized typical error of the estimate.

## Discussion

### Principal Findings

The current investigation was designed to assess the validity of 4 commercially available wrist-worn wearables for monitoring HR and EE while sitting or performing light-to-vigorous physical activity.

The following paragraphs outline our major findings.

For monitoring HR during sitting or walking/running up to 2.7 m/s or with a HR up to 167 bpm, the Apple Watch Series 4 demonstrated the highest validity (average 2.3 bpm deviation from the criterion measure), followed by the Polar Vantage V (5.9 bpm), Garmin Fenix 5 (9.1 bpm), and Fitbit Versa (13.3 bpm).

For monitoring HR when running at 3.6 m/s or faster, performing intermittent sprints, or with a HR of 153-177 bpm, the Apple Watch Series 4 again exhibited the highest validity (average 6.0 bpm deviation from the criterion measure), followed by the Polar Vantage V (8.5 bpm), Fitbit Versa (8.8 bpm), and Garmin Fenix 5 (11.0 bpm).

Overall, when measuring HR, the Apple Watch Series 4 was the most valid (average 3.9 bpm deviation from the criterion measure), followed by the Polar Vantage V (7.0 bpm), Garmin Fenix 5 (9.9 bpm), and Fitbit Versa (11.4 bpm).

The validity of HR monitoring by the Apple Watch Series 4 and Polar Vantage V tended to be influenced less by the exercise intensity than that with the Garmin Fenix 5 and Fitbit Versa.

On average, all 4 wearables were poor at monitoring EE at the tested intensities and durations. The Apple Watch Series 4 deviated from the criterion measure by 124 kcal/h (CV 21%), Polar Vantage V by 121 kcal/h (CV 20%), Garmin Fenix 5 by 131 kcal/h (CV 22%), and Fitbit Versa by 112 kcal/h (CV 19%): average for the different intensities, with extrapolation of the CV for the 5-minute measurements to 1 hour.

To the best of our knowledge, this is the first assessment of the validity of these specific wrist‑worn wearables. This is not surprising, since companies rarely rigorously validate new wearable models [20,21]. Comparison of our findings to earlier models requires caution, since it is not known whether the sensors or algorithms have been changed. However, such comparison might be of value to the manufacturers and to generally estimate if the parameters provided by the different manufacturers tend to be valid.

### Heart Rate Measurement

Previous comparison of earlier models of wrist-worn wearables sold by Apple, Polar, Garmin, and Fitbit at different intensities concluded that the Apple Watch Series 2 demonstrated the best validity for monitoring HR during exercise, followed by the Polar A380, Fitbit Blaze, Fitbit Charge 2, and Garmin Vivosmart HR, in that order, with absolute mean percentage errors of 4.1%, 19.5%, 21.1%, 21.4%, and 25.4%, respectively [32].

Another earlier comparison of the error rates of the Apple Watch (version not indicated), Fitbit Charge HR, and Garmin Forerunner 225 during light and vigorous running on a treadmill found that the Apple Watch displayed the highest validity (mean absolute percentage error of 1.1%-6.7%), followed by the Fitbit Charge HR (2.4%-17.0%) and Garmin Forerunner 225 (7.8%-24.4%) [33].

In addition, Thomson et al [34] validated HR measurements from the Fitbit Charge HR2 and Apple Watch of 30 young adults performing the Bruce Protocol and concluded that the relative error rates of the latter (2.4%-5.1%) were lower than for the Fitbit wearable (3.9%-13.5%) at all the investigated exercise intensities.

Thus, these previous and our present findings indicate that the wrist-worn wearables made by Apple Inc and Polar Electro Oy exhibit the highest validity for measuring HR during physical activity at different levels, followed by Garmin or Fitbit wearables. However, additional comparative studies with different populations and different activities are required.

### Energy Expenditure

The majority of the sTEE values for the EE values provided by all the wearables were large, very large, or extremely large. Even though the Apple Watch Series 4 had the best validity, its sTEE values ranged from moderate to very large, while those for the Polar Vantage V, Garmin Fenix 5, and Fitbit Versa ranged from moderate to extremely large, with no apparent dependency on exercise intensity. Since these error rates exceed acceptable levels of validity, we cannot determine whether the unpredictable arm movements associated with the intermittent multidirectional sprint protocol affected the validity.

Thus, utilization of these wearables by researchers monitoring EE during interventions designed to increase physical activity is likely to lead to flawed conclusions. They would not assist with enhancing physical activity or counteracting noncommunicable diseases and would instead endanger the trustworthiness of applying consumer grade wearables to improve health.

These findings of the poor validity of wrist-worn wearables for monitoring EE are in line with previous reports. Bai et al [35] found that the Apple Watch Series 1 had a smaller mean absolute percentage error (15.2%) when assessing EE than the Fitbit Wearable (32.9%), both when sedentary and during aerobic and light-to-vigorous physical activity [35].

Wahl et al [25] concluded that none of the 11 wrist-worn wearables they investigated, including devices from Garmin and Fitbit, should be used to monitor EE while performing activities of intensities similar to those investigated here. In a systematic review published in 2015, Evenson et al [21] stated that the validity of wearables for monitoring EE is low.

At the same time, when Kinnunen et al [36] aimed to assess the long-term validity of wrist-worn motion sensors for monitoring daily EE, they were able to explain as much as 85% of the variation in total EE (compared to the double-labelled water procedure) by including HR during weekly exercises in their analysis. This indicates the potential usefulness of wrist-worn wearables for estimating EE.

In a previous study that took age, gender, body mass, and HR into account, the correlation coefficient for predicting EE during 10 minutes of exercise could be as high as 0.913 with a mixed model [37]. Considering the considerable validity of HR measurements by wearables and the ability to incorporate all the information required into an appropriate algorithm, we believe that more precise estimation of EE by the wearables examined here should be feasible.

However, our findings and most of the available scientific literature indicate that the wearables investigated here should not be employed to estimate EE at these exercise intensities for the durations assessed. Here, we monitored EE for <5 minutes, since countries such as the United States or Australia promote such short periods of physical activity in their guidelines [38,39]. In this context, certain studies have demonstrated positive effects of even very brief vigorous exercise, such as walking up a staircase 3 times on 3 separate days each week for 6 weeks [40]. Whether these devices can be used to monitor EE reliably over longer time periods remains to be determined.

Our experiment involved Caucasians performing light-to-vigorous exercise on a treadmill under laboratory conditions, and extrapolation of our findings to other populations or settings (eg, cycling, rowing, strength training) must be performed with caution [22]. For example, skin color may influence assessment of HR by photoplethysmography. Moreover, since our participants performed either light or vigorous physical activity, we cannot draw conclusions about validity at moderate levels.

We wish to emphasize that our current findings only apply to the specific modes of the wearables we used (eg, the “indoor walking mode” for the Apple Watch) selected for the different physical activities and that other modes might give different results. The Apple Watch Series 4 and Polar Vantage V allow selection of more differentiated modes of activity (eg, the “indoor walking” and “indoor running” modes were selected on the Apple Watch for the corresponding activities) than the Garmin Fenix 5 and Fitbit Versa (for which the “Treadmill” mode was selected for all activities).

### Conclusions

For measuring HR while sitting or during light-to-vigorous physical activity, the Apple Watch Series 4 exhibited the best validity (ie, the smallest error rates), followed by the Polar Vantage V, Garmin Fenix 5, and Fitbit Versa, in that order. The Apple Watch Series 4 and Polar Vantage V can be used for valid HR measurements at the intensities tested, whereas HR acquired with the Garmin Fenix 5 and Fitbit Versa must be interpreted cautiously due to their higher rates of error.

None of these wrist-worn wearables should be used to monitor EE at the intensities and durations tested.

## Abbreviations

CV: coefficient of variation.
EE: energy expenditure.
HR: heart rate.
MET: metabolic equivalent.
sTEE: standardized typical error of the estimate.
WHO: World Health Organization.

## References

[1] (2018). World Health Organization.

[2] (2010). World Health Organization.

[3] World Health Organization (2020). World Health Organization.

[4] (2018). World Health Organization.

[5] Redenius N, Kim Y, Byun W (2019). Concurrent validity of the Fitbit for assessing sedentary behavior and moderate-to-vigorous physical activity. BMC Med Res Methodol.

[6] Trost SG (2001). Objective measurement of physical activity in youth: current issues, future directions. Exerc Sport Sci Rev.

[7] Seiler S (2010). What is best practice for training intensity and duration distribution in endurance athletes?. Int J Sports Physiol Perform.

[8] Swain DP, Abernathy KS, Smith CS, Lee SJ, Bunn SA (1994). Target heart rates for the development of cardiorespiratory fitness. Medicine & Science in Sports & Exercise.

[9] Donnelly JE, Blair SN, Jakicic JM, Manore MM, Rankin JW, Smith BK (2009). Appropriate Physical Activity Intervention Strategies for Weight Loss and Prevention of Weight Regain for Adults. Medicine & Science in Sports & Exercise.

[10] KOEHLER K, BRAUN H, DE MARÉES M, FUSCH G, FUSCH C, SCHAENZER W (2011). Assessing Energy Expenditure in Male Endurance Athletes. Medicine & Science in Sports & Exercise.

[11] Waldrop MM (2016). The chips are down for Moore's law. Nature.

[12] Thompson WR (2018). WORLDWIDE SURVEY OF FITNESS TRENDS FOR 2019. ACSMʼs Health & Fitness Journal.

[13] Gal R, May AM, van Overmeeren EJ, Simons M, Monninkhof EM (2018). The Effect of Physical Activity Interventions Comprising Wearables and Smartphone Applications on Physical Activity: a Systematic Review and Meta-analysis. Sports Med Open.

[14] Düking Peter, Achtzehn S, Holmberg H, Sperlich B (2018). Integrated Framework of Load Monitoring by a Combination of Smartphone Applications, Wearables and Point-of-Care Testing Provides Feedback that Allows Individual Responsive Adjustments to Activities of Daily Living. Sensors (Basel).

[15] Techniker Krankenkasse.

[16] World Health Organization.

[17] (2019). Deutscher Bundestag.

[18] Sperlich B, Holmberg H (2017). Wearable, yes, but able…?: it is time for evidence-based marketing claims!. Br J Sports Med.

[19] (2019). IDC.

[20] Düking Peter, Hotho A, Holmberg H, Fuss FK, Sperlich B (2016). Comparison of Non-Invasive Individual Monitoring of the Training and Health of Athletes with Commercially Available Wearable Technologies. Front Physiol.

[21] Evenson KR, Goto MM, Furberg RD (2015). Systematic review of the validity and reliability of consumer-wearable activity trackers. Int J Behav Nutr Phys Act.

[22] Düking Peter, Fuss FK, Holmberg H, Sperlich B (2018). Recommendations for Assessment of the Reliability, Sensitivity, and Validity of Data Provided by Wearable Sensors Designed for Monitoring Physical Activity. JMIR Mhealth Uhealth.

[23] Düking Peter, Born Dennis-Peter, Sperlich Billy (2016). The SpeedCourt: Reliability, Usefulness, and Validity of a New Method to Determine Change-of-Direction Speed. Int J Sports Physiol Perform.

[24] Vogler AJ, Rice AJ, Gore CJ (2010). Validity and reliability of the Cortex MetaMax3B portable metabolic system. J Sports Sci.

[25] Wahl Y, Düking Peter, Droszez A, Wahl P, Mester J (2017). Criterion-Validity of Commercially Available Physical Activity Tracker to Estimate Step Count, Covered Distance and Energy Expenditure during Sports Conditions. Front Physiol.

[26] Hermand E, Cassirame J, Ennequin G, Hue O (2019). Validation of a Photoplethysmographic Heart Rate Monitor: Polar OH1. Int J Sports Med.

[27] Schubert M, Clark A, De La Rosa A (2018). The Polar OH1 Optical Heart Rate Sensor is Valid during Moderate-Vigorous Exercise. Sports Med Int Open.

[28] Hopkins W (2017). Spreadsheets for analysis of validity and reliability. Sportscience.

[29] Khushhal A, Nichols S, Evans W, Gleadall-Siddall D, Page R, O'Doherty A, Carroll S, Ingle L, Abt G (2017). Validity and Reliability of the Apple Watch for Measuring Heart Rate During Exercise. Sports Med Int Open.

[30] Hopkins W (2016). Validity thresholds and error rates for test measures used to assess individuals. Proceedings of the 21st Annual Congress of the European College of Sport Science.

[31] Ainsworth B E, Haskell W L, Whitt M C, Irwin M L, Swartz A M, Strath S J, O'Brien W L, Bassett D R, Schmitz K H, Emplaincourt P O, Jacobs D R, Leon A S (2000). Compendium of physical activities: an update of activity codes and MET intensities. Med Sci Sports Exerc.

[32] BOUDREAUX BD, HEBERT EP, HOLLANDER DB, WILLIAMS BM, CORMIER CL, NAQUIN MR, GILLAN WW, GUSEW EE, KRAEMER RR (2018). Validity of Wearable Activity Monitors during Cycling and Resistance Exercise. Medicine & Science in Sports & Exercise.

[33] Dooley EE, Golaszewski NM, Bartholomew JB (2017). Estimating Accuracy at Exercise Intensities: A Comparative Study of Self-Monitoring Heart Rate and Physical Activity Wearable Devices. JMIR Mhealth Uhealth.

[34] Thomson EA, Nuss K, Comstock A, Reinwald S, Blake S, Pimentel RE, Tracy BL, Li K (2019). Heart rate measures from the Apple Watch, Fitbit Charge HR 2, and electrocardiogram across different exercise intensities. J Sports Sci.

[35] Bai Y, Hibbing P, Mantis C, Welk GJ (2018). Comparative evaluation of heart rate-based monitors: Apple Watch vs Fitbit Charge HR. J Sports Sci.

[36] Kinnunen H, Häkkinen Keijo, Schumann M, Karavirta L, Westerterp KR, Kyröläinen Heikki (2019). Training-induced changes in daily energy expenditure: Methodological evaluation using wrist-worn accelerometer, heart rate monitor, and doubly labeled water technique. PLoS One.

[37] Keytel L, Goedecke J, Noakes T, Hiiloskorpi H, Laukkanen R, van der Merwe L, Lambert E (2005). Prediction of energy expenditure from heart rate monitoring during submaximal exercise. J Sports Sci.

[38] (2019). The Department of Health, Australian Government.

[39] (2018). US Department of Health and Human Services.

[40] Jenkins EM, Nairn LN, Skelly LE, Little JP, Gibala MJ (2019). Do stair climbing exercise "snacks" improve cardiorespiratory fitness?. Appl Physiol Nutr Metab.

